# The origin of patient-derived cancer organoids from pathologically undiagnosed specimen in patients with pancreatobiliary cancers

**DOI:** 10.1007/s13402-024-01026-5

**Published:** 2024-12-17

**Authors:** Bomi Kim, Jiho Park, Hee Young Na, Sinwoo Park, Jeonghwa Jin, Kwangrok Jung, Jong-Chan Lee, Jin-Hyeok Hwang, Minseok Seo, Jaihwan Kim

**Affiliations:** 1 Department of Internal Medicine, Seoul National University Bundang Hospital, Seoul National University College of Medicine, Seongnam, Korea; 2 Next & Bio Inc., Seoul, Korea; 3 Department of Pathology, Seoul National University Bundang Hospital, Seoul National University College of Medicine, Seongnam, Korea; 4 Department of Computer and Information Science, Korea University, Sejong, Korea

**Keywords:** Pancreatic cancer, Gallbladder cancer, Patient derived cancer organoid, Precision therapy, Single-cell RNA sequencing

## Abstract

**Purpose:**

Tissue confirmation of pancreatobiliary cancer is often difficult because of the location of the tumor and structure of the surrounding blood vessels. Patient-derived cancer organoids (PDCOs) reflect the genomic characteristics of individual cancers. Although diverse attempts to construct PDCOs for various pancreatobiliary cancer models are ongoing, no research results have yet confirmed the possibility of performing a precise diagnosis on PDCOs derived from pathologically negative patient samples.

**Methods:**

We obtained a total of nine samples, including pathologically negative samples, from four patients (three patients with pancreatic cancer and one patient with gallbladder cancer) using different tissue acquisition methods to establish PDCOs (success rate 75%).

**Results:**

We successfully verified whether the constructed PDCOs could represent the tissues of patients with pancreatobiliary cancer at each multi-omics level using tumor panel sequencing, single-cell RNA sequencing, hematoxylin and eosin, and immunohistochemical staining. PDCOs from pathologically negative samples showed expression patterns of malignant ductal cell-related biomarkers similar to those of other pathologically positive samples. Furthermore, the expression patterns at the single-cell level in PDCO from patients ultimately diagnosed with gallbladder cancer after surgery were different from those in patients with pancreatic cancer.

**Conclusion:**

Therefore, our study implicated the potential of PDCOs as diagnostic and research tools, including for case involving limited tissue samples. Based on these results, we anticipate that this could be extended to more advanced studies, such as drug sensitivity testing, through large-scale trials in the near future.

**Supplementary Information:**

The online version contains supplementary material available at 10.1007/s13402-024-01026-5.

## Introduction

Pancreatic and biliary tract cancer are the representative lethal malignancies [1–3], and sometimes it is difficult to differentiate between the two diseases. For most patients, systemic therapy is required because they were frequently diagnosed at advanced stages [1, 2]. Tissue confirmation is mandatory prior to systemic therapy [4, 5]. However, this is sometimes challenging for pancreatobiliary cancers mainly because of the tumor location and surrounding vessels. Endoscopic ultrasound-guided fine-needle aspiration and biopsy (EUS-FNA/B), and endobiliary biopsy during endoscopic retrograde cholangiopancreatography (ERCP) are the cornerstone techniques for tissue acquisition from pancreatobiliary cancers [6]. The yields for two techniques are reported to be 73–89% (EUS-FNA/B) and 29–81% (ERCP), often due to limited sample amounts [7, 8]. Consequently, pathologically negative malignancy results based on EUS-FNA/B or ERCP tissue acquisition are not uncommon.

Recently, organoids have been widely used in research on pathogenesis, drug screening, and personalized medicine [9]. Patient-derived cancer organoids (PDCOs), models which reflect a patient’s genetic features, have been extensively studied for their potential applications in cancer research [9]. In pursuit of precision medicine, efforts are underway to establish and analyze patient-specific PDCOs, moving away from classical xenograft models [10, 11]. Generally, PDCOs are generated from surgical specimens [11]. However, given the low resectability rate of pancreatobiliary cancer, it is imperative to produce PDCOs from biopsies to encompass diverse patient populations. The current success rates of PDCOs from biopsies are comparable to those from surgical samples, with advancements in culture techniques [12]. Nevertheless, research on organoids derived from biopsies with pathologically negative results for malignancy is limited, making it challenging to assess their value.

Various attempts have been made to verify whether PDCOs can technically represent parental tumors. Whole-genome [13], exome [14], and cancer panel sequencing [15] technologies, which can be verified at the DNA level and are considered the most stable among molecular indicators, have been widely applied. To examine the homogeneity between PDCOs and real human tissues at the RNA level, bulk RNA-sequencing technology [16], with multiple biologically replicated samples and single-cell RNA-sequencing (scRNA-seq) [17] which examines expression levels at the single-cell level, are being actively applied. Immunohistochemical (IHC) staining can be used to compare expression markers. Although various technologies have demonstrated consistency between PDCO and actual tissues in vivo based on markers at various omics levels, each technology has practical limitations. Therefore, cross-validation at the multi-omics level is necessary to demonstrate the practical applicability of the PDCO.

Based on this research need, we established PDCOs from patients with pancreatobiliary cancer, including pathologically negative or questionable results, and conducted multi-omics integrated verification to determine whether the established PDCOs could represent actual parental tumors. Furthermore, based on the constructed PDCOs, we examined the possibility of developing a technology that can precisely distinguish pancreatobiliary cancer cases with pathologically negative or suspicious results.

## Results

### Characteristic of samples

All four patients had suspected pancreatic cancer at the initial radiological tests; however, one patient (patient 128) was diagnosed with gallbladder cancer (GBC) after surgery (Table 1). Patient 124 initially underwent ERCP and EUS-FNA/B on the same day; however, the pathological results were negative for malignancy. The patient underwent an additional EUS-FNA/B, and the pathological results were suggestive of ductal adenocarcinoma. Patients 128 and 134 received ERCP and EUS-FNA/B in a same day, and only EUS-FNA/B was positive for adenocarcinoma. Endobiliary biopsy from patient 128 was negative for malignancy, and that from patient 134 was suspicious for adenocarcinoma. Patients 124, 128, and 134 underwent surgery with curative intent after neoadjuvant chemotherapy. Patient 116 underwent an upfront surgery after EUS-FNA/B. The pathological result from EUS-FNA/B was suggestive of ductal adenocarcinoma, and the surgical pathological finding was moderately differentiated adenocarcinoma (intraductal papillary mucinous neoplasm with associated invasive carcinoma).

**Table 1:** Characteristics of samples for organoids

Patients	Sex/Age	Malignancies	Sampling methods	Pathological results	Passage 0	Passage 5	Thawing	Establishment	Sequencing Batch	Compared specimen
124	F/72	PDAC	ERCP	Negative	O	O	O	Success	1	Surgical specimen
			EUS-FNB	Suspicious	O	O	O	Success	2	EUS-FNB
			EUS-FNB	Negative	O	O	X	Fail	3	NA
128	M/73	GBC	ERCP	Negative	O	O	O	Success	1	Surgical specimen
			EUS-FNB	Positive	O	O	O	Success	2	EUS-FNB
134	F/61	PDAC	EUS-FNB	Positive	O	O	O	Success	3	EUS-FNB
			ERCP	Suspicious	O	O	O	Success	3	Surgical specimen
116	F/73	PDAC	EUS-FNB	Suspicious	O	X	X	Fail	-	-
			Surgery	Positive	O	O	O	Success	1	Surgical specimen

*PDAC* pancreatic ductal adenocarcinoma, *GBC* gallbladder cancer, *ERCP* endoscopic retrograde cholangiopancreatography, *EUS-FNA/B* Endoscopic ultrasound guided fine needle aspiration and biopsy

### Establishment of PDCOs and characterizations

**Fig. 1:**
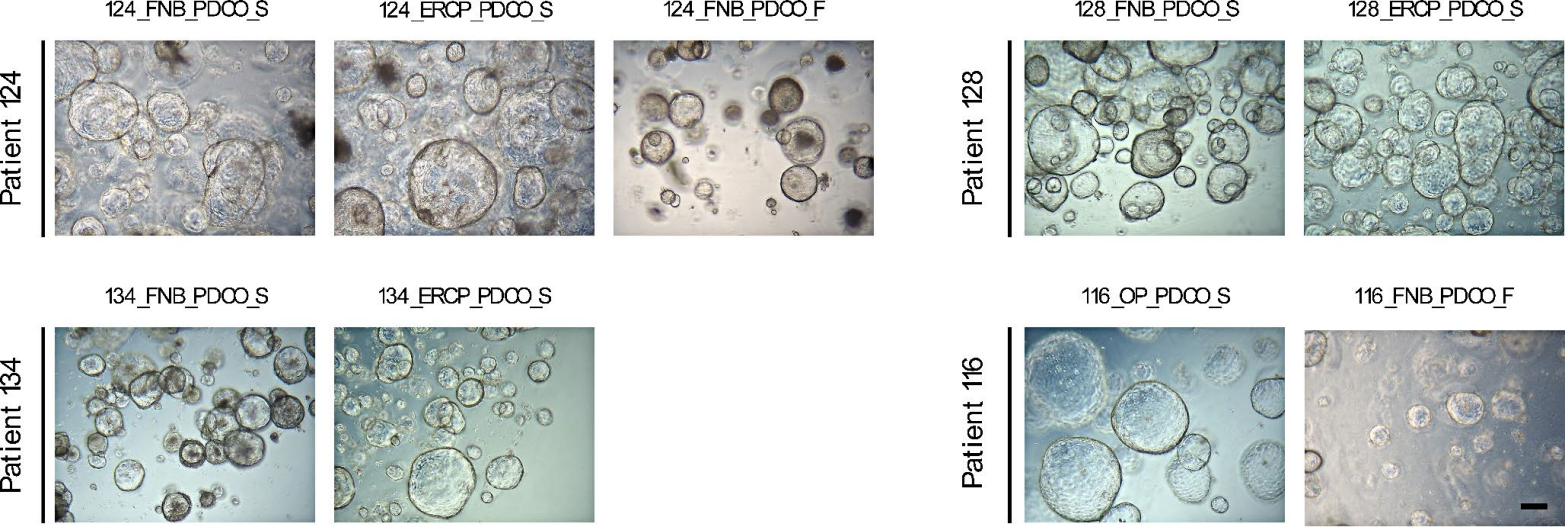
PDCO images from four patients. All nine samples collected by EUS-FNA/B, ERCP, or surgery from the four patients were primarily cultured. Each PDCO exhibited a characteristic cystic morphology and only eight PDCOs were successfully cultured until passage five. The PDCO derived from the FNB of patient 116 was cultured only up to passage 3. All images were captured during the primary culture period, specifically between passage 3 and passage 5. Scale bar represents 200 μm. *PDCO* patient-derived cancer organoid

We attempted to establish PDCOs from nine samples obtained from four patients, and successfully established seven of them (Fig. 1). Despite reports of methods that selectively inhibit the growth of normal organoids by removing niche factors and allowing only cancer organoids to proliferate, we used culture media containing Wnt3a and RSPO [18]. This approach was adopted to prevent the failure of organoid line establishment due to culture pressure, given the relatively limited initial sample size. Notably, we achieved successful organoid line establishment from samples obtained from a patient initially suspected to have pancreatic ductal adenocarcinoma (PDAC), but later confirmed to have GBC, even without utilizing a distinct culture medium composition for the GBC organoid.

Two failed attempts were confirmed based on different criteria (Table 1). The 116_FNB_PDCO_F case was categorized as an “expansion fail” case, as it was cultured up to passage 3 after initial seeding, but subsequently lost viability, failing to expand sufficiently for analysis and storage in cryo-vials. In contrast, 124_FNB_PDCO_F cells were cultured up to passage five, indicating successful expansion. However, it was categorized as a “thawing fail” case due to decreased viability after freezing and thawing.

Each PDCO exhibited a characteristic cystic morphology (Fig. 1). Regardless of the sample type, cancer type, and pathological results, all PDCOs displayed good viability, with successful establishment confirmed up to passage five. Even in the case of the two PDCOs that failed to establish, they maintained a cystic morphology in the primary culture. However, there were limitations to further analysis, as the cryo-stock preparation failed (116_FNB_PDCO_F) or viability decreased after thawing (124_FNB_PDCO_F).

### Comparison between PDCO and specimen in IHC analysis

**Fig. 2:**
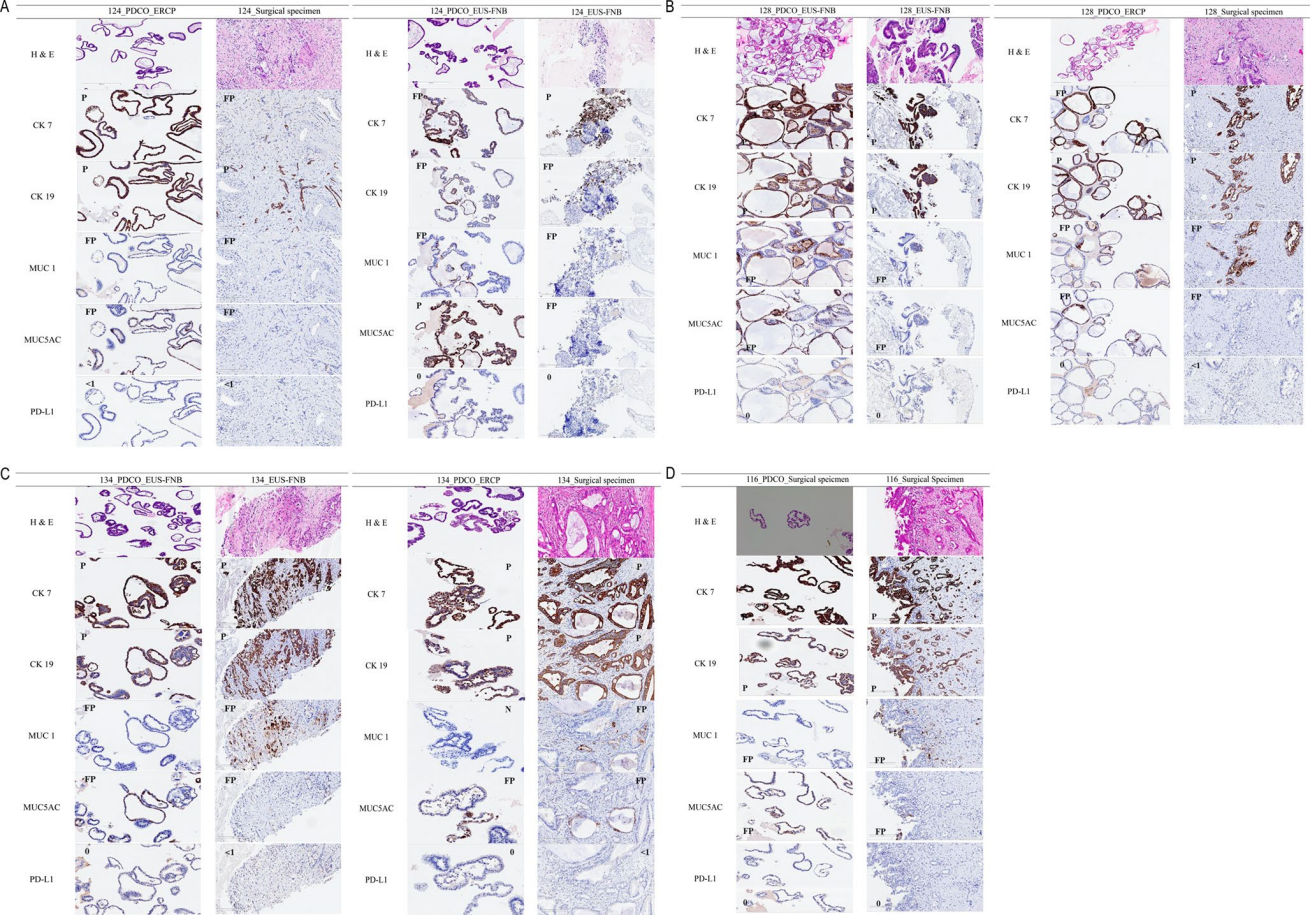
H&E and IHC staining images of PDCOs and pathological specimens. Hematoxylin and eosin (H&E) and immunohistochemical (IHC) staining were performed on the FFPE sections using specific antibodies. H&E staining revealed a similar microscopic morphology between PDCOs and paired pathological samples, characterized by malignant glandular epithelium forming a hollow lumen surrounded by tumor cells. The IHC staining results showed complete or nearly identical staining patterns in most sample pairs. *PDCO* patient-derived cancer organoid

In hematoxylin and eosin (H&E) staining, seven PDCOs showed a similar microscopic morphology to the paired pathological samples, which were characterized as malignant glandular epithelium, forming a hollow lumen surrounded by tumor cells (Fig. 2). For IHC staining, the results were identical in two pairs of samples (128_EUS-FNB and 116_surgical specimens) and nearly identical in five pairs of samples (124_ERCP, 124_EUS-FNB, 128_ERCP, 134_EUS-FNB, and 134_ERCP) (Table 2; Fig. 2). In particular, for PD-L1 staining, which is regarded as a potential predictive marker for immunotherapy, four of seven pairs (57.1%) were identical in tumor proportion score (TPS) and combined positive score (CPS), and two of seven (28.6%) were nearly identical (0 vs. <1%). However, the pair of 134_PDCO_ERCP and surgical specimens showed slight differences in CPS (0 vs. 5%).

**Table 2:** Comparison of PDCO and pathological specimen by IHC staining

Patients	Samples	CK7	CK19	MUC1	MUC5 AC	PD-L1 (22C3)-TPS (%)	PD-L1 (22C3)-CPS (%)
124	PDCO_ERCP	P	P	FP	FP	< 1	< 1
	Surgical specimen	FP	P	FP	FP	< 1	< 1
	PDCO_EUS-FNA/B	FP	FP	FP	P	0	0
	EUS-FNA/B	P	FP	FP	FP	0	0
128	PDCO_EUS-FNA/B	P	P	FP	FP	0	0
	EUS-FNA/B	P	P	FP	FP	0	0
	PDCO_ERCP	FP	P	FP	FP	0	0
	Surgical specimen	P	P	FP	FP	< 1	< 1
134	PDCO_EUS-FNA/B	P	P	FP	FP	0	0
	EUS-FNA/B	P	P	FP	FP	< 1	< 1
	PDCO_ERCP	P	P	N	FP	0	0
	Surgical specimen	P	P	FP	FP	< 1	5
116	PDCO_Surgical specimen	P	P	FP	FP	0	0
	Surgical specimen	P	P	FP	FP	0	0

*PDCO* patient-derived cancer organoids, *IHC* Immunohistochemistry, *ERCP* endoscopic retrograde cholangiopancreatography, *EUS-FNA/B* Endoscopic ultrasound guided fine needle aspiration and biopsy, *CK 7* Cytokeratin 7, *CK 19* Cytokeratin 19, *MUC1* Mucin1, *MUC 5AC* mucin 5AC, *TPS* tumor proportion score, *CPS* combined positive score, *P* positive, *FP* focal positive, *N* negative

### Comparison of genetic variant pattern between PDCOs and specimen

**Fig. 3:**
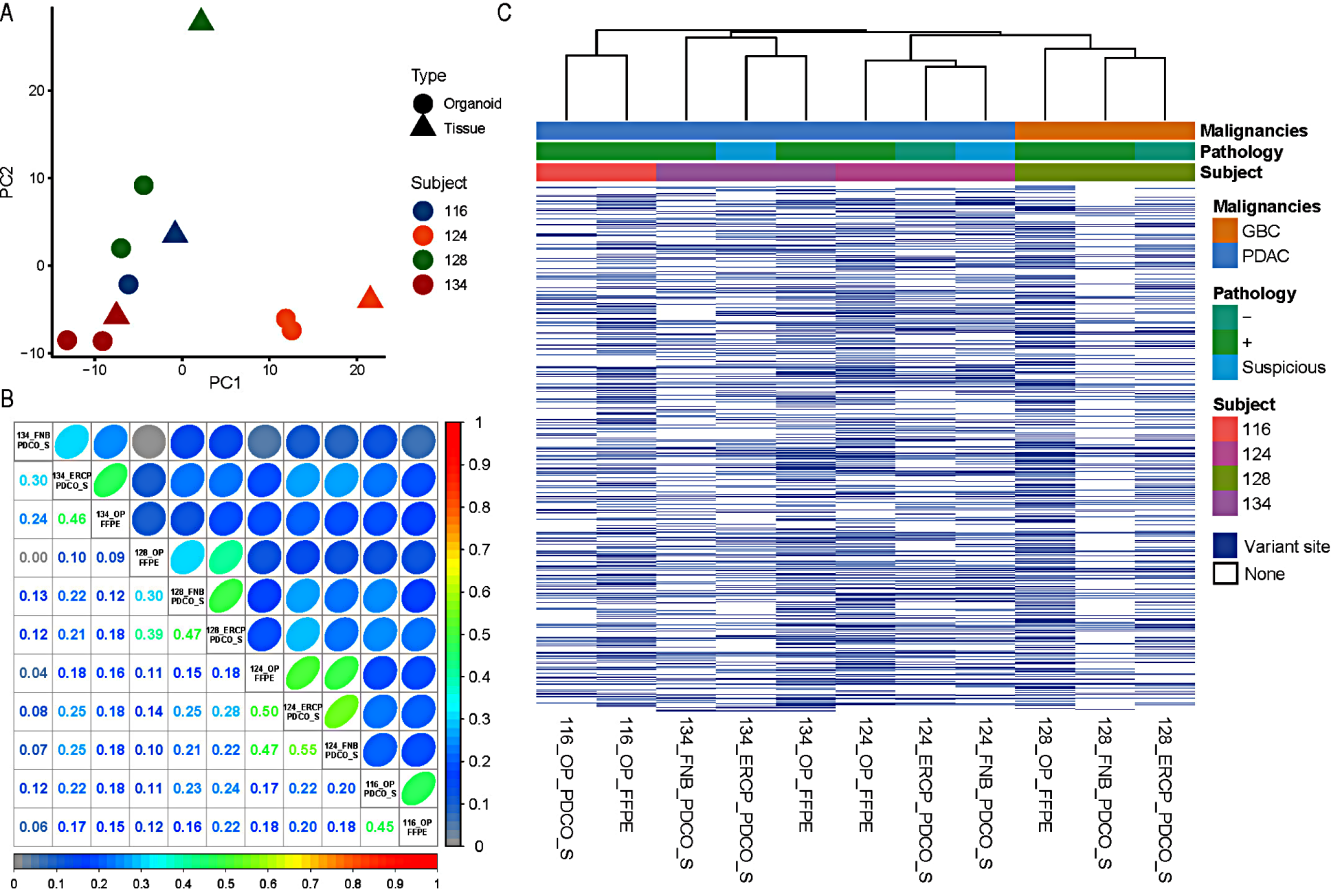
Comparative validation of PDCOs and actual pathological specimens at the DNA level based on unsupervised learning. **A** PCA-based dimension reduction plot of 4019 mutations called across a total of 11 samples, including PDCO and FFPE samples. **B** Pairwise correlations of 11 samples based on a total of 4019 variants. Samples originating from the same subject are highlighted with black squares. **C** Hierarchical clustering results and association analysis with various experimental conditions. *PDCO* patient-derived cancer organoid

We examined the mutation patterns of 11 samples (seven PDCOs and four surgical specimens from each patient) through tumor panel sequencing to investigate how well the PDCOs established at the DNA level matched the actual pathological specimens. After quality control, all variants found in the 11 samples were integrated, resulting in 4,019 mutations (Supplementary Data 1). Principal component analysis based on the mutation matrix verified that the variant pattern of each organoid was most similar to that of the actual surgical specimen, although there were certain differences between the PDCO and surgical specimens for each individual (Fig. 3A). Genetic correlations were calculated to examine the similarity of DNA sequences more objectively (Fig. 3B). An average variant similarity of 0.332 was observed for the two PDCOs derived from the patient 134 specimen. A high mutation identity of approximately 0.505 was observed in the PDCOs derived from patient 124. Similarly, we confirmed the similarity of the mutation patterns of 0.388 and 0.452 with the PDCOs in specimens from patients 128 and 116, respectively. The average mutation similarity between clusters was 0.163, indicating a heterogeneous pattern of genetic variants across subjects. On the other hand, the average mutation similarity within clusters derived from four patients was 0.419, which is direct evidence that PDCOs were derived from patient samples. These results directly support the fact that PDCOs can serve as representative models for the corresponding surgical specimens, although certain technical base sequence differences exist between PDCOs and surgical specimens. We further investigated the technical differences between PDCOs and formalin-fixed paraffin-embedded (FFPE) samples at the DNA level under various experimental conditions and found that this common difference was due to the number of called variants found in the FFPE specimens (Fig. 3C).

### Technical verification through characterization of cells derived from PDCOs

**Fig. 4:**
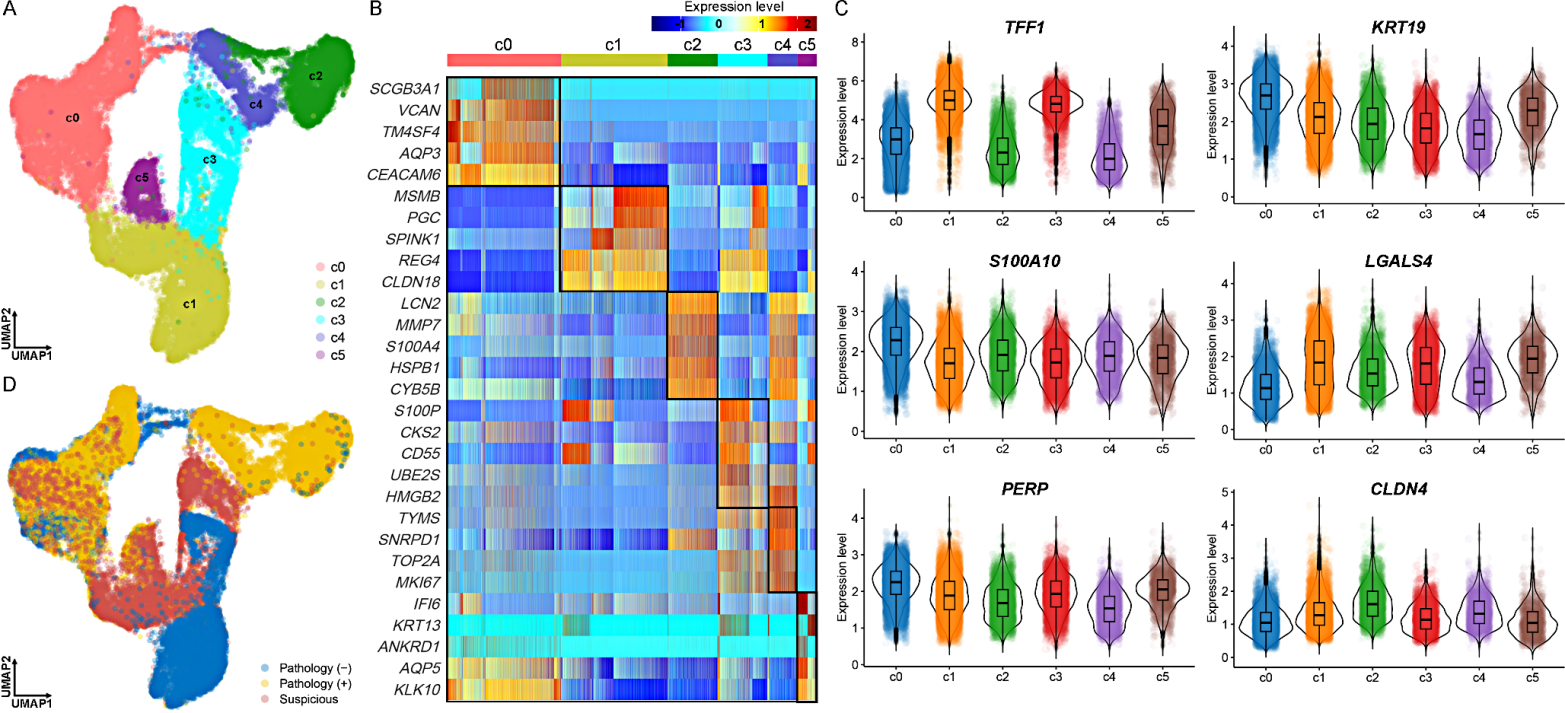
Technical validation and characterization of PDCO-derived single cells based on the scRNA-seq analysis. **A** UMAP dimensionality reduction plot showing heterogeneity of cells generated through scRNA-seq. Six subclusters, C0 to C5, were discovered based on the Silhouette score. **B** Expression patterns of subcluster specific markers through one-vs-others statistical hypothesis testing. In each cluster, the top five genes with the highest logFC among genes with adjP ≤ 0.05 were visualized. **C** Expression patterns of previously known representative ductal cell markers in each subcluster. **D** Stratified analysis based on pathological findings based on dimensionality reduction results of scRNA-seq. *PDCO* patient-derived cancer organoid, *scRNA-seq* single-cell RNA sequencing

We performed scRNA-seq to investigate the characteristics of cells grown on PDCOs and found that they were divided into six sub-clusters (Fig. 4A). Examination of the expression levels of transcripts specifically expressed in each subcluster revealed that most subclusters were not clearly distinguished, except for the C0 cluster (Fig. 4B). Therefore, we examined the expression levels of cell-specific markers to characterize all single cells generated from the PDCOs. We found that representative malignant ductal cell markers including *TFF1* [19], *KRT19* [20], and *S100A10* [21], were expressed at high levels in all cells derived from PDCOs generated under various conditions, indicating that most of the cells grown in PDCOs were malignant ductal cells (Fig. 4C). These results provide direct evidence that the PDCOs was successfully established for the desired experimental purposes. Based on the fact that ductal cell biomarkers are highly expressed in entire cells, we additionally attempted to stratify all single cells according to the actual patient’s pathological findings. Notably, cells derived from pathologically negative specimens also formed large clusters (Fig. 4D). Based on these results, we further established an additional hypothesized that markers of malignant ductal cells could be expressed despite negative pathological results.

### Exploring the potential of biomarkers at the single-cell transcriptome level through PDCO

**Fig. 5:**
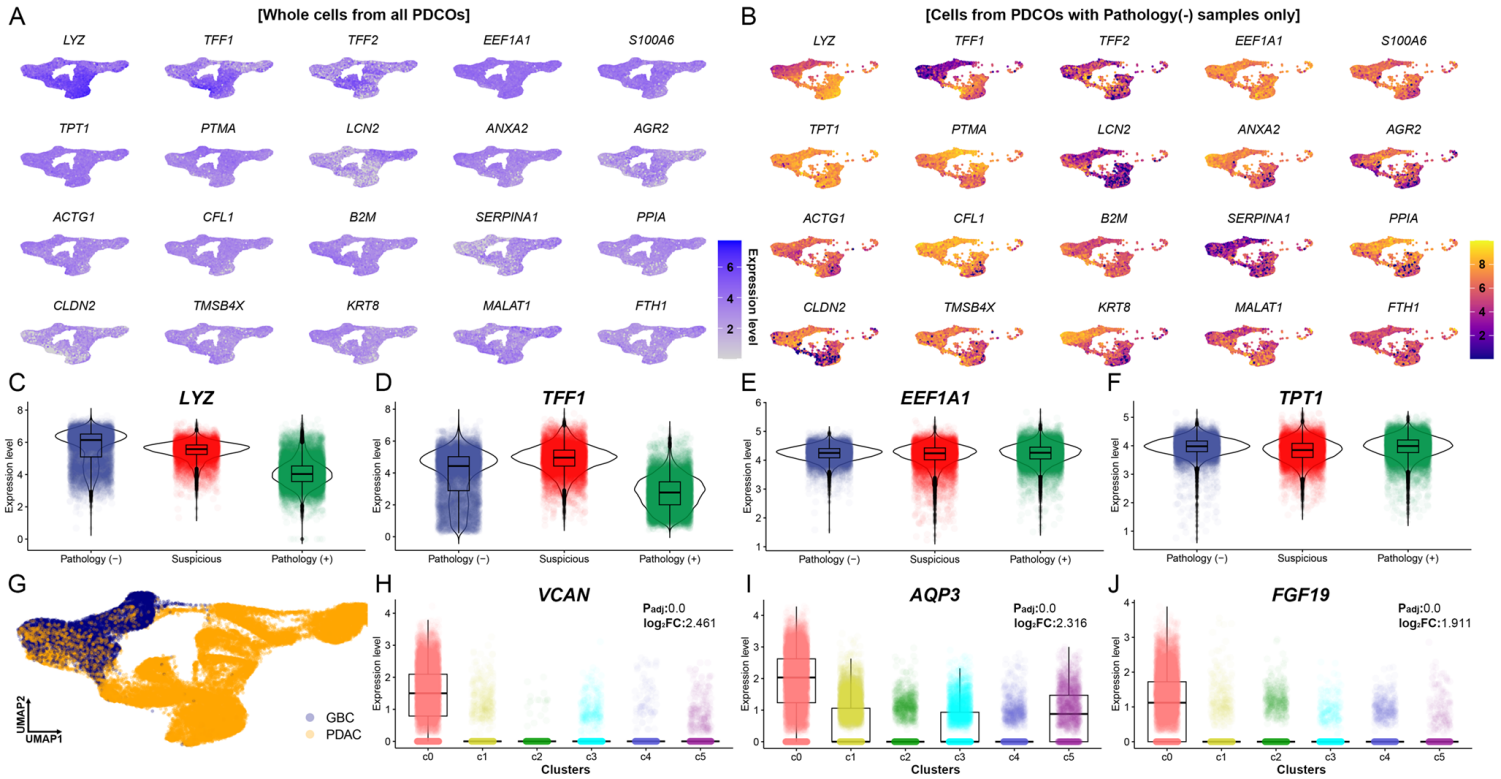
Exploring the possibility of early diagnosis of PDAC and/or GBC through identification of PDCO-derived transcriptome biomarkers at single cell level. **A** Top 20 genes highly expressed on average across whole cells. All discovered genes have been reported as representative diagnostic markers of PDAC. **B** These 20 genes were also expressed in cells from PDCOs from pathologically negative specimens. **C–F** Representative PDAC diagnostic markers, *LYZ*, *TFF1*, *EEF1A1*, and *TPT1*, show high expression levels even in cells derived from pathologically negative specimens. **G** UMAP dimensionality reduction plot stratified on the final diagnosis outcome. Most cells derived from GBC specimens belong to the C0 cluster. **H–J** Representative genes specifically expressed in C0 cluster at adjusted *P* ≤ 0.05. *GBC* gallbladder cancer, *PDAC* pancreatic ductal adenocarcinoma, *PDCO* patient-derived cancer organoid

**Fig. 6:**
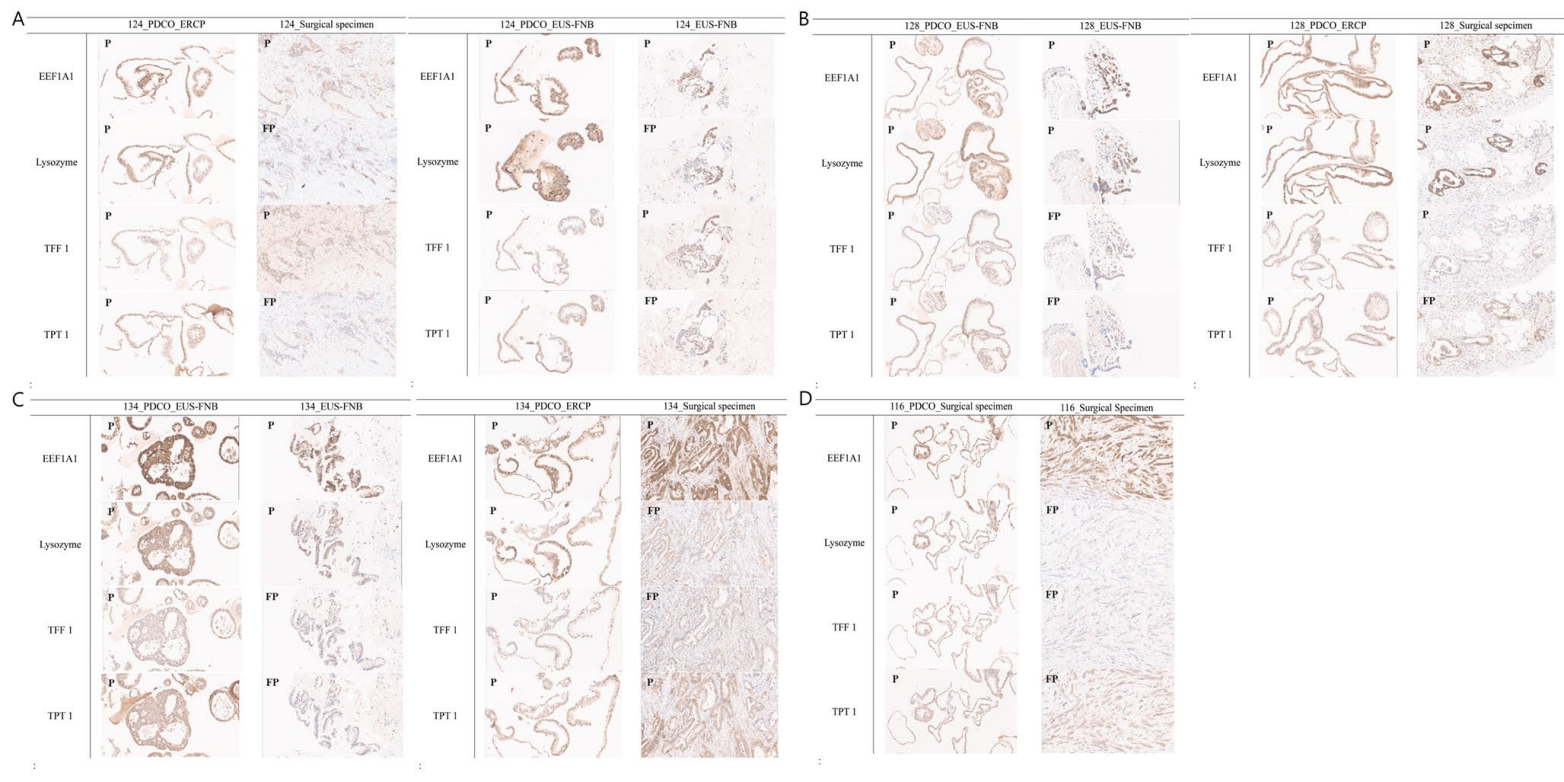
PDAC and/or malignant ductal cell related gene expression of PDCOs and pathological specimens by IHC staining images. Immunohistochemical (IHC) staining were performed on the FFPE sections using specific antibodies. The IHC staining results showed well expression of *EEF1A1*,* LYZ*,* TFF1*, and *TPT1* genes in both PDCOs and pathological specimens. *PDCO* patient-derived cancer organoid, *P* positive, *FP* focal positive

We further investigated the interesting phenomenon that transcripts were strongly expressed in all malignant ductal cells, despite the inclusion of pathologically negative samples. As a result of investigating for genes showing overrepresented expression patterns in whole cells derived from PDCOs, all of the top 20 transcripts were biomarkers related to PDAC and/or malignant ductal cells (Fig. 5A and Supplementary Data S2). Notably, genes, such as *LYZ*, *TFF1*, and *EFF1A1* were highly expressed in all cells, including cells derived from pathologically negative specimens (Fig. 5B and F) and those gene expression was validated in both PDCOs and pathological samples (Table 3; Fig. 6). In particular, the four proteins were all positive in PDCOs, but in the pathological samples, some were positive, and others were focal positive. This implies that relevant biomarkers discovered at the single-cell level via PDCO have the potential to be utilized in early diagnosis and may be missed in pathological decision-making.

**Table 3:** PDAC and/or malignant ductal cell related gene expression of PDCO and pathological specimen by IHC staining

Patients	Samples	EEF1A1	Lysozyme	TFF1	TPT1
124	PDCO_ERCP	P	P	P	P
	Surgical specimen	P	FP	P	FP
	PDCO_EUS-FNA/B	P	P	P	P
	EUS-FNA/B	P	FP	P	FP
128	PDCO_EUS-FNA/B	P	P	P	P
	EUS-FNA/B	P	P	FP	FP
	PDCO_ERCP	P	P	P	P
	Surgical specimen	P	P	P	FP
134	PDCO_EUS-FNA/B	P	P	P	P
	EUS-FNA/B	P	P	FP	FP
	PDCO_ERCP	P	P	P	P
	Surgical specimen	P	FP	FP	P
116	PDCO_Surgical specimen	P	P	P	P
	Surgical specimen	P	FP	FP	FP

*PDAC* pancreatic ductal adenocarcinoma, *PDCO* patient-derived cancer organoids, *IHC* Immunohistochemistry, *ERCP* endoscopic retrograde cholangiopancreatography, *EUS-FNA/B* Endoscopic ultrasound guided fine needle aspiration and biopsy, *P* positive, *FP* focal positive

We discovered that the final postoperative diagnosis in patient 128, who was suspected to have pancreatic cancer, was GBC. Stratified analysis was performed based on the final diagnosis and revealed that most cells in the C0 cluster (Fig. 4A and B), which were heterogeneous with other clusters, were derived from PDCOs with GBC (Fig. 5G). Genes, such as *VCAN*, *AQP3*, and *FGF19*, exhibited C0 cluster-specific expression patterns already identified through cluster-specific biomarker identification analysis, which is the most fundamental analysis of scRNA-seq that can distinguish cell heterogeneity, and are differentially expressed genes between GBC and PDAC (Fig. 5H and J).

## Discussion

We established seven PDCO lines from nine samples obtained from four patients, despite variable cancer types and pathological results, using the same culture medium containing growth factors and Wnt signaling pathway activators. The PDCOs made from such small amounts of samples were subjected to H&E and IHC staining, scRNA-seq, and DNA cancer panel analysis, showing the characteristics of carcinoma, regardless of the pathology results.

In this study, a notable achievement was the successful generation of organoids using small tissue samples obtained through ERCP and EUS-FNA/B. A recent meta-analysis also reported the comparability of patient-derived tumor organoids between EUS-guided biopsies and surgical specimens [12]. Compared to the meta-analysis that reported establishment success rates of 60%, 36%, and 62% from EUS-guided biopsies, percutaneous biopsies, and surgical specimens, respectively, our study reported that the overall establishment success rates were 77.8% and 75.0% from EUS-FNA/B and ERCP biopsies (except for one surgical sample). The high success rates of biopsies indicate that endoscopic tissue acquisition is a comparable method for the establishment of PDCOs with surgical samples, despite the inherent challenge posed by limited sample amounts.

We introduced two criteria to determine the success of PDCO establishment. One was the “expansion capability,” which assessed whether the organoids could be continuously cultured while maintaining viability up to passage 5. Despite the high initial success rate in primary cultures, the ability to maintain viability during long-term culture was considered separately. When the criterion ‘capable of culturing for more than 5 passages’ is applied, the success rate of establishing organoids decreases [22]. The other criterion was the “thawing test,” which assessed whether the organoids-maintained viability when re-cultured after freezing. These two criteria are crucial for determining whether established organoids can be continuously utilized for genetic analysis, drug sensitivity testing, and other purposes, thereby determining the actual value of the PDCO establishment. This study generated and analyzed PDCOs from a limited number of patients, therefore, the correlation between the pathological results of the biopsy samples and organoid establishment remains to be determined. We were able to establish organoids from samples judged as pathologically negative; although all pathologically negative samples were successful in the initial stage of primary culture, some organoids encountered issues with expansion or viability after thawing. Further attempts to establish PDCOs from more patient-derived negative pathological samples are required to clarify this.

We confirmed that *LYZ*, *TFF1*, and *TFF2* genes, which were overexpressed in ductal cells, were overexpressed in the same manner as ductal cell-related marker genes found in PDAC in previous human PDAC single-cell data analysis studies [23, 24]. These results were identical to those in this study (Fig. 5A and D). In previous single-cell analysis studies based on human PDAC organoids, these three genes also showed a pattern of overexpression in specific organoid samples, consistent with our results [25, 26]. Another single-cell analysis study using human PDAC organoids identified the *EEF1A1* gene as a marker [24, 27], consistent with our findings. The *EEF1A1* gene is known to interact with the *FBXO32* gene [28] to promote PDAC progression and contribute to tumor growth and metastasis [29]. The fact that the representative genes identified in the PDCOs in this study were found identically in actual PDAC patients is one of the pieces of evidence demonstrating the reliability of our results.

In this study, PDCOs without a pathological diagnosis of cancer showed the same results as those with a pathological diagnosis using H&E and IHC staining (Figs. 1 and 2), DNA mutation analysis (Fig. 3), and scRNA-seq (Fig. 4). Our results are similar with previous studies showing that PDCOs can capture the characteristics of the original tumor and serve as a tool for personalized medicine [30, 31]. Notably, the diagnosis of one patient changed from pancreatic cancer to GBC. The radiologic diagnosis was initially synchronous pancreatic cancer and GBC; however, the surgical specimen revealed that the GBC invaded the surrounding lymph nodes and focal pancreas. The cell type of this patient was intracholecystic tubulopapillary neoplasm with an associated adenocarcinoma, which is under the heading of “papillary adenocarcinoma” [32]. Notably, this study suggested that PDCO cells derived from a patient with GBC were enriched in the C0-cluster and could be distinguished from the malignant ductal cells of PDCOs derived from other patients with pancreatic cancer (Fig. 5G). We performed cluster-specific marker discovery analysis, which is fundamental for scRNA-seq data analysis of the C0-cluster, and found that three genes, *VCAN*, *AQP3*, and *FGF19*, were significantly upregulated in the C0-cluster (Fig. 5H and J). The *VCAN*, which is specifically expressed in the c0-cluster, was identified as overexpressed in myeloid-derived suppressor cells (MDSC) in a previous single-cell analysis study based on human gallbladder cancer [33]. The *VCAN*, translated into the CSPG2 protein, has been reported as a marker for the metastasis of various carcinomas, including bladder carcinoma [34, 35]. In addition, aquaporin 3 (*AQP3*) is an important regulator of the inflammatory response and a marker that can identify the effects of gallbladder damage [36, 37]. Furthermore, *FGF19* can promote the progression of GBC [38].

Considering these results, the detection of specific expression patterns of cells in PDCO derived from patients with GBC suggests the possibility that it can be employed for diagnosing GBC and/or predicting prognosis, where accurate diagnosis is practically difficult.

Our study has several limitations. First, the inclusion of GBC samples, originally thought to be pancreatic cancer, led to new findings; however, the sample size was too small for generalization. Second, the acquisition methods varied and included ERCP-guided forceps biopsy, EUS-FNA/B, and surgery. However, we did not find a different finding based on the sample acquisition method used in this study. We believe that these practical limitations can be naturally resolved through larger-sample experiments in the near future.

In conclusion, our study highlights the potential of PDCOs as valuable diagnostic and research tools in oncology, particularly in scenarios in which only small tissue samples are available. The consistency of the results obtained from PDCOs, regardless of the underlying pathology, holds promise for advancing our understanding of cancer biology and improving patient care through personalized treatment approaches.

## Methods

### Study population and acquisition method for samples

Pancreatic cancer samples were prospectively collected in 2021. Four patients were enrolled in this cohort and seven samples were collected. For tissue acquisition, three patients underwent EUS-FNA/B and ERCP, and one patient underwent surgery without a preoperative tissue diagnosis. All four patients underwent surgeries with curative intent. All EUS-FNA/B and ERCP procedures were performed by a skilled endoscopist (J.K.) at a single tertiary teaching hospital. Standard curvilinear array echoendoscopes (GF-UCT260, Olympus America, Central Valley, PA, USA) and duodenoscopes (TJF-240 or JF-240, Olympus Optical Co. Ltd., Tokyo, Japan) were used for all patients. EUS-FNA/B examinations were mostly performed using 22-gauge standard FNA/B needles (Expect needle, Boston Scientific Inc., Marlborough, MA, USA, or ClearTip needle, Finemedix, Daegu, Republic of Korea). This study was conducted in accordance with the guidelines of the Declaration of Helsinki and approved by the Institutional Review Board of Seoul National University Bundang Hospital (approval numbers: B-2006-621-304 and B-2302-812-302). Informed consent was obtained from all patients.

### Sample preparation and PDCOs culture

EUS-FNA, ERCP biopsy, and surgical samples in a 15-mL conical tube were washed and centrifuged at 400 g for 3 min. The supernatant was discarded and the tissue was minced with a surgical blade (Surgical blade No.10, Feather, Osaka, Japan). The minced tissue was transferred to a C-tube (Catalog no.130-093-237, Miltenyi Biotec, Bergisch Gladbach, Germany) and digested enzymatically and mechanically with gentleMACS™ Tissue Dissociators (Catalog no.130-096-427, Miltenyi Biotec). The digested cells were washed with basal medium (advanced DMEM, 1% Penicillin/Streptomycin, and 1% HEPES), embedded in growth factor-reduced Matrigel, and placed in 12-well plates. After 37℃ incubation for 15 min, culture media was added. The culture media were as previously reported, with some modifications [39]. In brief, it was supplemented with the following growth factors: 100 ng/mL of Wnt3a, 1 ug/mL of RSPO1, and 50 ng/mL of EGF. The culture medium was replaced every 3 days, and the organoids were cultured until at least passage 5 before being stored in cryovials.

### H&E and IHC staining

FFPE blocks were constructed from both PDCOs and pathological samples, including biopsy and surgically resected specimens. For the morphological comparison, H&E and IHC staining were performed after sectioning FFPE blocks at a 4-µm thickness. For IHC, anti-CK7 (OV-TL 12/30, 1:600, Agilent, Santa Clara, CA, USA), anti-CK19 (RCK108, 1:150, Agilent), anti-MUC 1 (Ma695, 1:100, Leica Biosystems, Buffalo Grove, IL, USA), anti-MUC5AC (CLH2, 1:100, Leica Biosystems) and anti-PD-L1 (22C3, 1:50, Agilent), anti-EEF1A1 (DF6156, 1:500, Affinity Biosciences, Cincinnati, OH), anti-lysozyme (DF7890, 1:1000, Affinity Biosciences), anti-TFF1 (DF6619, 1:100, Affinity Biosciences), anti-TPT1 (DF7343, 1:400, Affinity Biosciences) antibodies were used to investigate the characteristics of tumor cells in pathology samples and PDCOs. IHC for all antibodies, except anti-PD-L1, was performed according to validated protocols using an automated immunostainer (Ventana, Tuscon, AZ, USA). For PD-L1, FFPE blocks were immunostained using the EnVision FLEX visualization system on a Dako Autostainer Link 48 platform (Agilent), according to the manufacturer’s instructions. The H&E and IHC slides were digitally scanned and analyzed using the Aperio ImageScope software v.12.4.6. (Leica Biosystems). For CK7, CK19, MUC1, and MUC5AC, EEF1A1, lysozyme, TFF1, and TPT1 staining results were scored into three semi-quantitative categories: negative, no staining; focal positive, positive in < 90% of tumor cells; and positive, positive in ≥ 90% of tumor cells. PD-L1 expression was evaluated in both tumor and immune cells. For tumor cells, complete and/or partial circumferential membranous staining with any intensity was considered PD-L1 positive, while membrane and/or cytoplasmic staining of mononuclear inflammatory cells (lymphocytes and macrophages) within tumor nests and adjacent supporting stroma at any intensity was counted. Finally, the TPS and CPS were measured. TPS was defined as the percentage of viable tumor cells relative to all viable tumor cells [40, 41]. CPS was defined as the number of PD-L1 positive cells (both tumor and immune cells) divided by the total number of viable tumor cells and multiplied by 100. The maximum CPS value In case of FNA/B specimens with “positive” pathology results, PDCOs were compared with FNA/B samples. In the case of FNA/B or ERCP specimens with “negative” pathology results, PDCOs were compared with surgical specimens because of the lack of tumor cells in FNA/B or ERCP specimen FFPE blocks. When pathological results were “suspicious for adenocarcinoma” in FNA/B or ERCP specimens, PDCOs were compared with FNA/B samples in which tumor cells were sufficient. A comparison with surgical specimens was performed on FNA/B or ERCP samples with few tumor cells, which did not allow for additional IHC studies. All morphological comparisons of H&E and IHC staining results were performed by an experienced pathologist (H.Y.N.).

### Tumor panel sequencing

Fragmented DNA (0.2 μm) was prepared to construct libraries with the SureSelect Cancer CGP assay included 679 genes (Agilent) using manufacturer’s protocol and analyzed by the Illumina NovaSeq 6000 (Theragen Bio, Seongnam, Korea) according to the manufacturer’s recommendations. Cutadapt was used to remove adapter sequences from the raw sequencing data and generate clean reads with an average sequencing quality of Q20 or better [42]. The cleaned reads were mapped to the hg38 human reference genome using a BWA aligner [43]. Subsequently, deduplication and base quality score corrections were performed using the GATK Base Recalibrator pipeline. Mutect2 was used to identify variants based on a panel of Korean variant information from the Korean Reference Genome Database [44–46]. Finally, SnpEff was used to annotate identified variants [47].

### Homogeneity analysis of variant pattern between PDCOs and pathological samples

To determine whether the mutation patterns of PDCOs and actual pathological samples were consistent at the DNA level, genetic analysis was performed based on the VCF files of each sample obtained through tumor panel sequencing. First, we aggregate all variant files (.vcf files) derived from GATK and Muteck2 across all samples, and then removed variants whose filter columns did not belong to germline, somatic, haplotype, or panel_of_normals. A matrix (number of samples by number of whole variants) was then constructed according to whether a mutation was found at a specific site, depending on its location in the genome of each sample. Based on the constructed matrix, principal component analysis was performed to visualize the similarity between the PDCOs and variant patterns of the pathological specimens. In addition, hierarchical clustering was performed with the dissimilarity metric (1-absolute value of Pearson’s correlation) and the complete linkage method based on the similarity of the mutation pattern in the whole genome between samples.

### ScRNA-seq analysis

PDCOs were collected and dissociated into single cells by incubating with TrypLE for 5 min at 37 °C. Single cells were counted using a Luna-II automated cell counter (Logos Biosystems), each sample was labeled with an appropriate sample multiplexing antibody, Single-Cell multiplex kit - Human (BD Bioscience, Franklin Lakes, NJ, USA), and up to three samples were pooled together in equal numbers of 20,000 cells (60,000 cells in total) and loaded on a microwell cartridge of the BD Rhapsody Express system (BD Biosciences). Single-cell whole-transcriptome libraries were prepared according to the manufacturer’s instructions using the BD Rhapsody WTA Reagent kit (BD Biosciences). Libraries were sequenced using an Illumina HiSeq X™ in Macrogen (Seoul, Korea). Fastq files were processed using BD WTA Multiplex Rhapsody Analysis Pipeline v1.9.1 (https://bitbucket.org/CRSwDev/cwl/src/master/). In this step, FASTQ reads were demultiplexed, mapped to the human GRCh38 reference genome (STAR aligner v2.5.2b), and gene/barcode matrices were performed [48]. Finally, the raw counts were adjusted using a distribution-based error correction developed by BD Biosciences. These count matrices were loaded in Seurat v4.3.0.1 for downstream analysis [49]. We used only cells with a unique feature count > 400 and mitochondrial percentage < 40% in the downstream analysis. After normalization using *the NormalizeData*() method implemented in Seurat, 2,000 highly variable features were selected through variance-stabilizing transformation, and principal component analysis was performed. Harmony (v1.1.0) was employed to correct batch effects that can occur when performing the analysis by integrating multiple Seurat objects [50]. The UMAP algorithm was used to visualize cell subtype expression patterns. The average silhouette score was used to determine the optimal number of subclusters. To identify genes showing subcluster-specific expression patterns, the *FindAllMarkers*() function implemented in Seurat was used with the default option. Finally, *AverageExpression*(), implemented in Seurat, was used to identify genes with the highest average expression in all cells or cells derived from samples under specific conditions. In this study, a false discovery rate-adjusted P-value of 0.05 or less after adjustment was used as the statistical significance threshold [51].

## Electronic supplementary material

Below is the link to the electronic supplementary material.

Supplementary Material 1

Supplementary Material 2

## Data Availability

No datasets were generated or analysed during the current study.
