# Supplementary material for: The origin of patient-derived cancer organoids from pathologically undiagnosed specimen in patients with pancreatobiliary cancers
Source: Cell Oncol (Dordr). 2024 Dec 17;48(2):523–35. doi: 10.1007/s13402-024-01026-5 (PMC11996933; doi:10.1007/s13402-024-01026-5)
Supplement: Supplementary file 2 — Supplementary Material 2 [file 13402_2024_1026_MOESM2_ESM.docx]

Supplementary Data S2. Top 20 genes highly expressed on average in single cells derived from PDCOs

| **geneSymbol** | **WholeCells** | **Pathology(-)** | **Suspicious** | **Pathology(+)** | **GBC** | **PDAC** | **C0** | **C1** | **C2** | **C3** | **C4** | **C5** | **Ref** |
| --- | --- | --- | --- | --- | --- | --- | --- | --- | --- | --- | --- | --- | --- |
| *LYZ* | 1241.22 | 1931.80 | 1300.72 | 823.64 | 888.72 | 1314.39 | 98.55 | 483.91 | 78.32 | 336.62 | 59.54 | 184.27 | [1] |
| *TFF1* | 453.81 | 432.47 | 680.69 | 246.74 | 275.52 | 459.24 | 25.93 | 196.02 | 16.37 | 139.20 | 12.72 | 63.57 | [2] |
| *TFF2* | 445.88 | 234.96 | 745.46 | 308.01 | 287.31 | 457.64 | 39.77 | 115.41 | 31.16 | 123.56 | 23.18 | 112.80 | [2] |
| *EEF1A1* | 414.20 | 391.47 | 422.14 | 392.33 | 369.60 | 409.24 | 72.29 | 69.87 | 75.88 | 65.62 | 64.71 | 65.83 | [3] |
| *S100A6* | 336.02 | 239.25 | 485.49 | 277.21 | 319.81 | 322.37 | 58.37 | 61.84 | 38.16 | 73.35 | 32.82 | 71.48 | [4] |
| *TPT1* | 304.54 | 314.65 | 275.24 | 311.02 | 285.16 | 300.89 | 56.12 | 54.28 | 54.36 | 44.72 | 47.67 | 47.38 | [5] |
| *PTMA* | 233.33 | 207.48 | 282.12 | 213.21 | 246.98 | 221.98 | 46.12 | 26.08 | 30.36 | 54.44 | 47.22 | 29.12 | [6] |
| *LCN2* | 184.27 | 114.89 | 86.46 | 214.40 | 106.96 | 192.94 | 13.65 | 7.77 | 74.91 | 8.08 | 53.66 | 26.20 | [7] |
| *ANXA2* | 183.80 | 175.59 | 199.86 | 189.23 | 166.47 | 182.65 | 38.14 | 23.25 | 28.69 | 28.70 | 28.34 | 36.69 | [8] |
| *AGR2* | 155.94 | 142.20 | 201.91 | 115.96 | 156.83 | 141.18 | 44.86 | 33.24 | 10.68 | 37.82 | 9.60 | 19.74 | [9] |
| *ACTG1* | 154.17 | 150.06 | 162.70 | 146.00 | 168.93 | 149.02 | 31.75 | 25.78 | 25.38 | 26.50 | 26.77 | 17.98 | [10] |
| *CFL1* | 149.86 | 141.12 | 156.48 | 152.16 | 147.57 | 147.95 | 27.32 | 21.91 | 23.10 | 26.40 | 26.78 | 24.33 | [11] |
| *B2M* | 145.68 | 151.76 | 153.64 | 171.35 | 123.70 | 152.73 | 21.29 | 28.61 | 21.20 | 21.30 | 17.70 | 35.58 | [12] |
| *SERPINA1* | 137.74 | 119.82 | 114.36 | 137.29 | 100.21 | 140.95 | 10.46 | 23.78 | 33.23 | 19.13 | 24.13 | 27.01 | [13] |
| *PPIA* | 133.95 | 116.78 | 134.76 | 132.25 | 120.66 | 130.86 | 22.67 | 15.79 | 24.26 | 23.20 | 28.60 | 19.43 | [14] |
| *CLDN2* | 122.94 | 126.67 | 108.53 | 119.85 | 123.02 | 124.04 | 24.76 | 13.94 | 19.00 | 19.69 | 20.12 | 25.43 | [15] |
| *TMSB4X* | 109.67 | 113.33 | 102.07 | 122.39 | 104.24 | 111.17 | 22.85 | 17.58 | 20.78 | 12.90 | 17.31 | 18.25 | [16] |
| *KRT8* | 109.21 | 125.82 | 103.63 | 118.14 | 133.92 | 108.04 | 35.04 | 13.31 | 12.92 | 13.65 | 13.17 | 21.11 | [17] |
| *MALAT1* | 107.72 | 108.96 | 85.25 | 124.56 | 189.34 | 111.50 | 14.18 | 18.05 | 26.78 | 9.49 | 23.16 | 16.06 | [18] |
| *FTH1* | 107.44 | 88.71 | 80.50 | 124.68 | 78.62 | 107.29 | 12.89 | 13.47 | 30.58 | 10.89 | 23.44 | 16.17 | [19] |

All values ​​are log2 normalized expression values ​​called by Seurat's *AverageExpression*(). WholeCells refers to the average expression level of the corresponding transcript in all cells. *Pathology(-)* to *Pathology(+)* columns represent the average expression level in cells obtained according to the pathological outcome of each sample. GBC and PDAC represent the average expression level in cells obtained according to the final clinical diagnosis result, and C0 to C5 represent the average expression level for each sub-cluster of cells.

**Reference**

1. Wang, Y., et al., *Single-cell analysis of pancreatic ductal adenocarcinoma identifies a novel fibroblast subtype associated with poor prognosis but better immunotherapy response.* Cell discovery, 2021. **7**(1): p. 36.

2. Ebert, M., et al., *Induction of TFF1 gene expression in pancreas overexpressing transforming growth factor α.* Gut, 1999. **45**(1): p. 105-111.

3. Liu, S., et al., *METTL13 methylation of eEF1A increases translational output to promote tumorigenesis.* Cell, 2019. **176**(3): p. 491-504. e21.

4. Vimalachandran, D., et al., *High nuclear S100A6 (Calcyclin) is significantly associated with poor survival in pancreatic cancer patients.* Cancer research, 2005. **65**(8): p. 3218-3225.

5. You, Y., et al., *MicroRNA-216b-5p functions as a tumor-suppressive RNA by targeting TPT1 in pancreatic cancer cells.* Journal of Cancer, 2017. **8**(14): p. 2854.

6. Prudova, A., et al., *TAILS N-terminomics and proteomics show protein degradation dominates over proteolytic processing by cathepsins in pancreatic tumors.* Cell reports, 2016. **16**(6): p. 1762-1773.

7. Gomez-Chou, S.B., et al., *Lipocalin-2 promotes pancreatic ductal adenocarcinoma by regulating inflammation in the tumor microenvironment.* Cancer research, 2017. **77**(10): p. 2647-2660.

8. Takahashi, H., et al., *High expression of Annexin A2 is associated with DNA repair, metabolic alteration, and worse survival in pancreatic ductal adenocarcinoma.* Surgery, 2019. **166**(2): p. 150-156.

9. Dumartin, L., et al., *ER stress protein AGR2 precedes and is involved in the regulation of pancreatic cancer initiation.* Oncogene, 2017. **36**(22): p. 3094-3103.

10. Tang, Y., et al., *Actin gamma 1 is a critical regulator of pancreatic ductal adenocarcinoma.* Saudi Journal of Gastroenterology: Official Journal of the Saudi Gastroenterology Association, 2022. **28**(3): p. 239.

11. Castillo, L., et al., *MCL-1 antagonism enhances the anti-invasive effects of dasatinib in pancreatic adenocarcinoma.* Oncogene, 2020. **39**(8): p. 1821-1829.

12. Liu, C., et al., *Overexpression of B2M and loss of ALK7 expression are associated with invasion, metastasis, and poor-prognosis of the pancreatic ductal adenocarcinoma.* Cancer Biomarkers, 2015. **15**(6): p. 735-743.

13. Atay, S., *Integrated transcriptome meta-analysis of pancreatic ductal adenocarcinoma and matched adjacent pancreatic tissues.* PeerJ, 2020. **8**: p. e10141.

14. Wang, S., et al., *High expression level of peptidylprolyl isomerase A is correlated with poor prognosis of liver hepatocellular carcinoma.* Oncology letters, 2019. **18**(5): p. 4691-4702.

15. Kesaraju, S., et al., *Claudin-2 limits pancreatitis development through regulating tight junction-controlled pancreatic ductal transport.* bioRxiv, 2023: p. 2023.09. 01.555960.

16. Grüner, B.M., et al., *MALDI imaging mass spectrometry for in situ proteomic analysis of preneoplastic lesions in pancreatic cancer.* PloS one, 2012. **7**(6): p. e39424.

17. Pistoni, L., et al., *Associations between pancreatic expression quantitative traits and risk of pancreatic ductal adenocarcinoma.* Carcinogenesis, 2021. **42**(8): p. 1037-1045.

18. Liu, J.-H., et al., *Expression and prognostic significance of lncRNA MALAT1 in pancreatic cancer tissues.* Asian Pacific Journal of Cancer Prevention, 2014. **15**(7): p. 2971-2977.

19. Park, J.M., et al., *A case–control study in Taiwanese cohort and meta-analysis of serum ferritin in pancreatic cancer.* Scientific Reports, 2021. **11**(1): p. 21242.
